# Long-Term Management of Pediatric Chronic Diseases: Improving Quality of Life and Reducing Hospital Admissions in Children With Asthma, Cystic Fibrosis, Diabetes, and Epilepsy

**DOI:** 10.7759/cureus.76529

**Published:** 2024-12-28

**Authors:** Muhammad Shah Nawaz Khan, Shah Fahad, Maithem Haider, Syed Asad Hasan, Sania Chaudhry, Talha Amjad

**Affiliations:** 1 Physiology, HBS Medical and Dental College, Islamabad, PAK; 2 Biochemistry, HBS Medical and Dental College, Islamabad, PAK; 3 Anatomy, HBS Medical and Dental College, Islamabad, PAK

**Keywords:** family engagement, hospital admissions, pediatric chronic diseases, quality of life, technology-driven monitoring

## Abstract

Background: Children who suffer from long-term illnesses, including asthma, cystic fibrosis, diabetes, or epilepsy, sometimes struggle to manage their ailments, which affects their quality of life and how often they use healthcare services.

Objective: This study aimed to explore comprehensive long-term management strategies for children with asthma, cystic fibrosis, diabetes, and epilepsy, with a focus on enhancing quality of life and reducing hospital admissions.

Methodology: A prospective cohort research was conducted involving 480 children, divided into four groups: 120 children with asthma, 120 children with cystic fibrosis, 120 children with diabetes, and 120 children with epilepsy. Participants were evaluated at baseline and at several follow-ups (3, 6, 12, and 24 months) across a 24-month period. Structured surveys, including questions on treatment adherence and quality of life metrics, as well as checks of medical records to monitor hospital admissions, were used to gather data. To investigate changes in hospital admission rates and quality of life scores over time, statistical analyses were performed, including paired t-tests. Statistical significance was defined as a p-value of less than 0.05.

Results: Quality of life scores improved significantly for all groups, with asthma patients demonstrating the most significant increase of 12.53 ± 3.51 points, rising from a baseline score of 62.54 ± 14.03 to 75.07 ± 10.52 (p < 0.001). Hospital admissions also declined substantially, particularly in the asthma group, which reduced from 4.51 ± 2.07 to 2.06 ± 1.37 (p < 0.001). High adherence rates were observed among patients, with 85 (70.83%) in asthma, 90 (75.00%) in cystic fibrosis, 95 (79.17%) in diabetes, and 92 (76.67%) in epilepsy. Additionally, patient satisfaction scores were notably high, averaging 78.02 ± 10.07 in asthma, 80.03 ± 9.52 in cystic fibrosis, 82.21 ± 8.05 in diabetes, and 79.15 ± 9.03 in epilepsy across the different disease categories.

Conclusion: Children with chronic illnesses have a much higher quality of life and fewer hospital admissions when family engagement techniques and technology-driven monitoring are used.

## Introduction

Chronic diseases in children represent a significant healthcare issue that impacts global healthcare systems and people's quality of life [1]. Unlike acute illnesses, juvenile chronic disorders such as asthma, cystic fibrosis, diabetes, and epilepsy need ongoing care and provide unique treatment problems since they must be managed for years or even a lifetime [2]. The fact that chronic disorders often lead to repeated hospitalizations and place a major cost on families and healthcare systems underscores the need for effective long-term treatment strategies [3].

Asthma affects millions of children globally and is often caused by uncontrolled environmental factors [4]. Cystic fibrosis, although less prevalent, presents serious treatment challenges, such as the need for regular physical therapy and medications to prevent potentially lethal lung infections [5]. Type 1 diabetes may be difficult for children and their caregivers due to the necessity of insulin delivery, dietary restrictions, and continuous blood glucose monitoring [6]. Managing epilepsy requires careful medication therapy and continuous monitoring to reduce seizure risks and improve daily functioning, even if the severity and frequency of episodes vary [7].

These chronic diseases affect academic performance and emotional well-being in addition to emergency medical treatment [8]. The stigmatization of many children with chronic diseases, their limited participation in school activities, and their limitations in social connections may lead to anxiety, hopelessness, and poor self-esteem [9]. To treat these aspects of chronic disease, a holistic approach that addresses the child's physical and mental health needs is required [10].

While current treatment approaches often focus on symptom reduction and issue avoidance, there is increasing recognition that improving quality of life should be the primary aim [11]. Programs that use family participation, multidisciplinary approaches, and technology-driven monitoring systems are showing promise as a means of reducing hospitalizations and enhancing overall health [12]. However, challenges exist regarding the implementation and accessibility of these technologies, particularly in underfunded settings. This study aimed to explore comprehensive long-term management strategies for children with asthma, cystic fibrosis, diabetes, and epilepsy, with a focus on enhancing quality of life and reducing hospital admissions.

## Materials and methods

Study design and setting

This study was a longitudinal, observational cohort conducted at the HBS Medical and Dental College in Islamabad from January 2022 to December 2023.

Inclusion and exclusion criteria

Children aged 8-16 with asthma, cystic fibrosis, type 1 diabetes, or epilepsy requiring routine follow-up were included, provided their families consented to participation and follow-up evaluations. Exclusion criteria included children with unrelated severe chronic illnesses, a history of missed appointments or treatment non-adherence, or unwillingness to provide informed consent.

Sample size

The World Health Organization's (WHO) algorithm for cohort studies was used to determine the study's sample size in order to guarantee sufficient power to identify statistically significant changes in hospital admissions and quality of life. A 95% confidence level, a *z*-score of 1.96 with a 5% (0.05) margin of error, was established. In order to optimize the necessary sample size in situations when the actual proportion is unknown, a cautious estimate of the percentage of pediatric chronic disease hospitalizations was set at 0.5 (50%). Using the formula $n = \frac{z^{2}.p.(1-p)}{E2}$, the initial calculation yielded a sample size of approximately 384 participants. A dropout rate of 20% was considered to account for potential dropouts, leading to an adjusted sample size of 480 participants.

Data collection

Baseline data included demographics, medical history, and initial quality of life scores measured using the Pediatric Quality of Life Inventory (PedsQL) [13]. Follow-up evaluations conducted quarterly included PedsQL and Strengths and Difficulties Questionnaire (SDQ) scores [14], alongside caregiver insights, school attendance, and social involvement. Behavioral and medical metrics such as hospitalizations, adherence, and treatment adjustments were also documented.

Interventions were supported by multidisciplinary teams comprising pediatricians, nurses, dietitians, and mental health specialists who developed individualized management plans. Family engagement strategies and technology-driven monitoring, such as mobile health apps, were incorporated to enhance adherence and provide real-time feedback. Families were trained to use mobile health applications to monitor children's health, medication adherence, and symptoms. These apps allowed caregivers to track progress, report health updates, and receive reminders for medications and appointments.

Additionally, structured caregiver training sessions were conducted to familiarize parents with telehealth tools and technology platforms. These sessions included tutorials on using apps for medication tracking, symptom reporting, and communication with healthcare providers. Caregivers participated in hands-on practice and Q&A forums to ensure they were confident in using these tools.

While these technology-driven strategies enhanced adherence and engagement, challenges such as limited access to smartphones or internet connectivity for some families were noted. Moreover, some caregivers found certain app features complex, requiring additional training and support. Despite these challenges, technology integration significantly contributed to continuous monitoring and feedback.

Hospitalization refers to the admission of a patient to the hospital for medical treatment or procedures that require an overnight stay or longer. This includes both planned and unplanned admissions related to the child's chronic condition or any complications arising from it.

Rehospitalization refers to any subsequent admission of the same patient to the hospital after being discharged, typically within a set period (e.g., 30 days), either due to the recurrence of the condition or complications related to the initial hospitalization.

Quality of life and psychosocial well-being scoring

Quality of life was assessed using the PedsQL, which measures four domains: physical, emotional, social, and school functioning [13]. Each domain is scored from 0 to 100, with higher scores indicating better quality of life. Psychosocial well-being was assessed using the SDQ, which evaluates emotional symptoms, conduct problems, hyperactivity, peer relationships, and prosocial behavior [14]. The validity and reliability of these tools have been well established for use in pediatric populations [13-15].

Statistical analysis

The statistical analysis for this study was conducted using IBM SPSS Statistics for Windows, Version 26 (Released 2019; IBM Corp., Armonk, New York). Descriptive statistics were computed for baseline demographics, medical history, and initial quality of life scores. A one-way ANOVA was used to compare continuous variables, including age, medical history (duration of diagnosis and previous hospital admissions), quality of life scores, and psychosocial well-being scores across the four conditions (asthma, cystic fibrosis, diabetes, and epilepsy). Categorical variables, such as gender, socioeconomic status, and treatment adherence, were analyzed using chi-square tests (χ^2^). Changes over time in quality of life scores, psychosocial well-being, hospital admission rates, and treatment adherence were analyzed using repeated measures ANOVA, with pairwise comparisons conducted to assess the significance of differences at each follow-up time point. A p-value of less than 0.05 was considered statistically significant. Post-hoc analyses were performed for all tests to explore differences between individual groups where appropriate.

Ethical approval

The Institutional Review Board (IRB) reviewed and approved the study protocol. Written informed consent was obtained from parents or guardians, and assent was sought from children when appropriate. Patient privacy and data confidentiality were upheld throughout the study.

## Results

In the study, children with asthma, cystic fibrosis, type 1 diabetes, and epilepsy had mean ages of 11.86 ± 2.43, 12.52 ± 2.61, 12.07 ± 2.58, and 12.24 ± 2.52 years at baseline, respectively (Table 1). The gender distribution among the study population was relatively balanced, with 51.67% (n = 62) of children with asthma, 47.50% (n = 57) with cystic fibrosis, 53.33% (n = 64) with diabetes, and 49.16% (n = 59) with epilepsy being male, indicating nearly equal gender representation across the conditions. Regarding socioeconomic background, approximately 41-44% of patients from each condition came from low socioeconomic backgrounds (n = 50-53), 36-40% from medium socioeconomic backgrounds (n = 43-48), and 18-20% from high socioeconomic backgrounds (n = 22-25), showing a modest variation in socioeconomic status across the groups. In terms of medical history, the average time from diagnosis varied by condition: children with asthma had a mean diagnosis duration of 2.84 ± 1.97 years, while those with cystic fibrosis had a longer mean duration of 4.02 ± 2.36 years. Hospital admission rates were highest for asthma (4.51 ± 2.07 admissions), followed by cystic fibrosis, epilepsy, and diabetes, which had the lowest rate of 2.13 ± 1.72 admissions. Psychosocial well-being ratings ranged from 55.04 ± 11.58 for asthma to 60.01 ± 12.06 for diabetes, while the initial quality of life scores were lowest for asthma (62.54 ± 14.03) and highest for diabetes (67.51 ± 14.53).

**Table 1:** Baseline assessment of demographic data, medical history, and initial quality of life scores Age: The mean age of participants across different groups. The differences in age were assessed using a one-way ANOVA (F-test), with no statistically significant differences across groups (p = 0.674). Gender: The distribution of male and female participants in each group, analyzed using the chi-square test (χ^2^), showing no significant gender differences across groups (p = 0.787). Socioeconomic status: Participants were categorized based on their family's reported socioeconomic status. The data were analyzed using the chi-square test (χ^2^), with no significant differences (p = 0.732). Medical history: The average number of years participants had been diagnosed with their respective conditions, analyzed using ANOVA (F = 18.72), revealing significant differences across groups (p < 0.001). The average number of hospital admissions due to the chronic disease, with significant differences between groups (F = 10.27, p < 0.001). Initial quality of life scores: The quality of life score was measured using the Pediatric Quality of Life Inventory (PedsQL) scale (range 0-100), where higher scores indicate better quality of life. The data were analyzed using ANOVA (F = 2.68), with significant differences between groups (p = 0.049). The psychosocial well-being score was measured using the Strengths and Difficulties Questionnaire (SDQ), where higher scores reflect better psychosocial functioning. The data analysis showed significant differences (F = 3.17, p = 0.024).

Characteristic		Asthma (n = 120)	Cystic Fibrosis (n = 120)	Diabetes (n = 120)	Epilepsy (n = 120)	Test Statistic (t/χ^2^/F)	P-value
Age (years)	Mean ± SD	11.86 ± 2.43	12.52 ± 2.61	12.07 ± 2.58	12.24 ± 2.52	F = 0.51	0.674
Gender	Male	62 (51.67%)	57 (47.50%)	64 (53.33%)	59 (49.16%)	χ^2^ = 1.75	0.787
	Female	58 (48.33%)	63 (52.50%)	56 (46.67%)	61 (50.84%)		
Socioeconomic status	Low	50 (41.67%)	52 (43.33%)	49 (40.83%)	53 (44.17%)	χ^2^ = 1.30	0.732
	Middle	45 (37.50%)	46 (38.33%)	48 (40.00%)	43 (35.83%)		
	High	25 (20.83%)	22 (18.34%)	23 (19.17%)	24 (20.00%)		
Medical history	Duration of diagnosis (years)	2.84 ± 1.97	4.02 ± 2.36	3.21 ± 2.03	3.54 ± 2.16	F = 18.72	<0.001
	Previous hospital admissions	4.51 ± 2.07	3.14 ± 1.59	2.13 ± 1.72	3.35 ± 1.28	F = 10.27	<0.001
Initial quality of life scores	Quality of life score (scale 0-100)	62.54 ± 14.03	66.02 ± 16.05	67.51 ± 14.53	66.28 ± 15.07	F = 2.68	0.049
	Psychosocial well-being score (scale 0-100)	55.04 ± 11.58	59.53 ± 13.02	60.01 ± 12.06	59.02 ± 12.57	F = 3.17	0.024

All pediatric chronic conditions showed significant improvements in quality of life scores over a 24-month period, with patients with asthma seeing increases from 62.54 ± 14.03 at baseline to 75.07 ± 10.52, those with cystic fibrosis from 66.02 ± 16.05 to 75.81 ± 12.17, those with diabetes from 67.51 ± 14.53 to 78.46 ± 9.51, and those with epilepsy from 66.28 ± 15.07 to 76.38 ± 10.09 (Table 2). The scores for psychosocial well-being also increased, with diabetes improving the most, reaching 67.78 ± 7.46. All categories had a decrease in hospital admissions, although diabetes had the lowest average (1.37 ± 1.08). Diabetes had the greatest rate of treatment adherence (79.17% high adherence), whereas asthma had the lowest rate (70.83% high adherence). While caregiver stress was lowest in diabetes (63.32 ± 12.52) and greatest in asthma (65.51 ± 14.57), patient satisfaction ratings varied from 70.21 in asthma to 74.11 in diabetes.

**Table 2:** Longitudinal follow-up of quality of life, hospital admissions, treatment adherence, adjustments, and patient-caregiver outcomes across pediatric chronic diseases Quality of life and psychosocial well-being scores: Measured on a 0-100 scale, higher scores indicate better outcomes. The scores are reported at 3, 6, 12, and 24 months. Frequency of hospital admissions: Average number of admissions over the two-year period, with lower values indicating fewer hospitalizations. Treatment adherence: Categorized into high, moderate, or low adherence based on the percentage of patients following prescribed regimens. Number of adjustments in treatment plans: Average number of treatment modifications over the two-year follow-up. Patient satisfaction: Measured on a 0-100 scale, higher values reflect greater satisfaction with care. Caregiver stress: Measured on a 0-100 scale, with higher scores indicating higher stress levels. Test statistics: F-values for ANOVA and χ^2^-values for chi-square tests compare differences between groups. A p-value < 0.05 indicates statistical significance. Statistical significance: Significance is denoted by p < 0.05, highlighting meaningful differences across the conditions. Test statistics: ANOVA (F) and chi-square test (χ^2^) were used to compare the differences between the groups for each characteristic. A p-value of less than 0.05 is considered statistically significant, indicating a meaningful difference between the conditions (asthma, cystic fibrosis, diabetes, and epilepsy).

Characteristic		Asthma (n = 120)	Cystic Fibrosis (n = 120)	Diabetes (n = 120)	Epilepsy (n = 120)	Test Statistic	P-value
Quality of life score (scale 0-100)	Follow-up 1 (3 months)	65.53 ± 14.52	68.09 ± 15.13	70.19 ± 12.54	69.51 ± 13.46	F = 1.22	0.312
	Follow-up 2 (6 months)	68.19 ± 13.78	70.62 ± 14.21	73.12 ± 11.47	72.13 ± 12.52	F = 1.95	0.125
	Follow-up 3 (12 months)	72.32 ± 12.46	73.53 ± 13.57	76.59 ± 10.13	75.27 ± 11.31	F = 3.03	0.033
	Follow-up 4 (24 months)	75.07 ± 10.52	75.81 ± 12.17	78.46 ± 9.51	76.38 ± 10.09	F = 1.25	0.293
Psychosocial well-being score (scale 0-100)	Follow-up 1 (3 months)	58.09 ± 11.02	60.89 ± 10.53	61.79 ± 10.31	60.59 ± 11.54	F = 1.16	0.327
	Follow-up 2 (6 months)	60.13 ± 10.21	62.17 ± 9.52	63.21 ± 9.54	62.87 ± 10.23	F = 1.64	0.186
	Follow-up 3 (12 months)	63.43 ± 9.17	64.54 ± 8.51	65.63 ± 8.74	65.88 ± 9.12	F = 0.99	0.401
	Follow-up 4 (24 months)	65.88 ± 8.16	66.31 ± 7.57	67.78 ± 7.46	67.05 ± 8.11	F = 0.53	0.662
Frequency of hospital admissions (2 years)	Mean ± SD	2.06 ± 1.37	1.53 ± 1.12	1.37 ± 1.08	1.84 ± 1.42	F = 3.88	0.01
Treatment adherence	High adherence (%)	85 (70.83%)	90 (75.00%)	95 (79.17%)	92 (76.67%)	χ^2^ = 2.34	0.504
	Moderate adherence (%)	30 (25.00%)	25 (20.83%)	20 (16.67%)	25 (20.83%)		
	Low adherence (%)	5 (4.17%)	5 (4.17%)	5 (4.17%)	3 (2.50%)		
Number of adjustments in treatment plans	Mean ± SD	1.27 ± 0.83	1.52 ± 0.91	1.34 ± 0.72	1.45 ± 0.89	F = 1.56	0.208
Overall satisfaction score (scale 0-100)	Mean ± SD	70.21 ± 12.01	72.34 ± 11.15	74.11 ± 10.17	73.51 ± 12.57	F = 1.36	0.258
Caregiver stress score (scale 0-100)	Mean ± SD	65.51 ± 14.57	64.19 ± 13.25	63.32 ± 12.52	64.54 ± 14.07	F = 0.45	0.718

**Figure 1:**
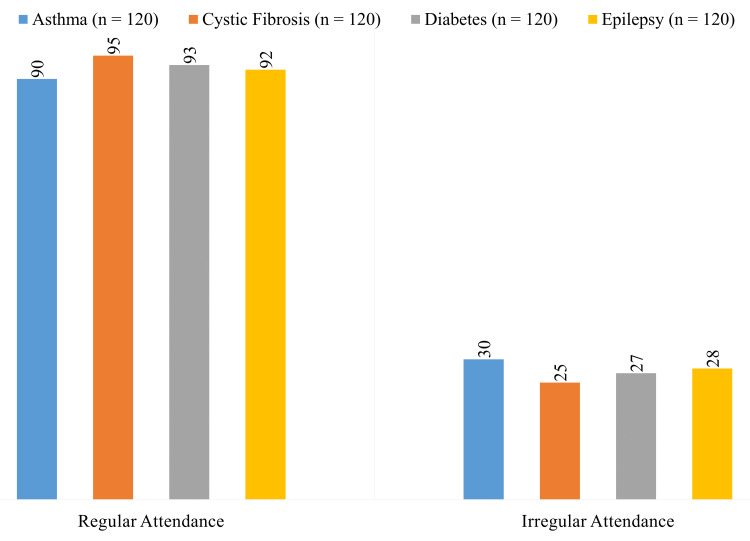
School attendance rates among pediatric patients with asthma, cystic fibrosis, diabetes, and epilepsy.

According to Figure 1, individuals with cystic fibrosis had the greatest rates of regular school attendance (n = 95), followed by those with diabetes (n = 93), epilepsy (n = 92), and asthma (n = 90). Patients with asthma (n = 30) were most likely to have irregular attendance, followed by those with diabetes (n = 27), epilepsy (n = 28), and cystic fibrosis (n = 25).

**Figure 2:**
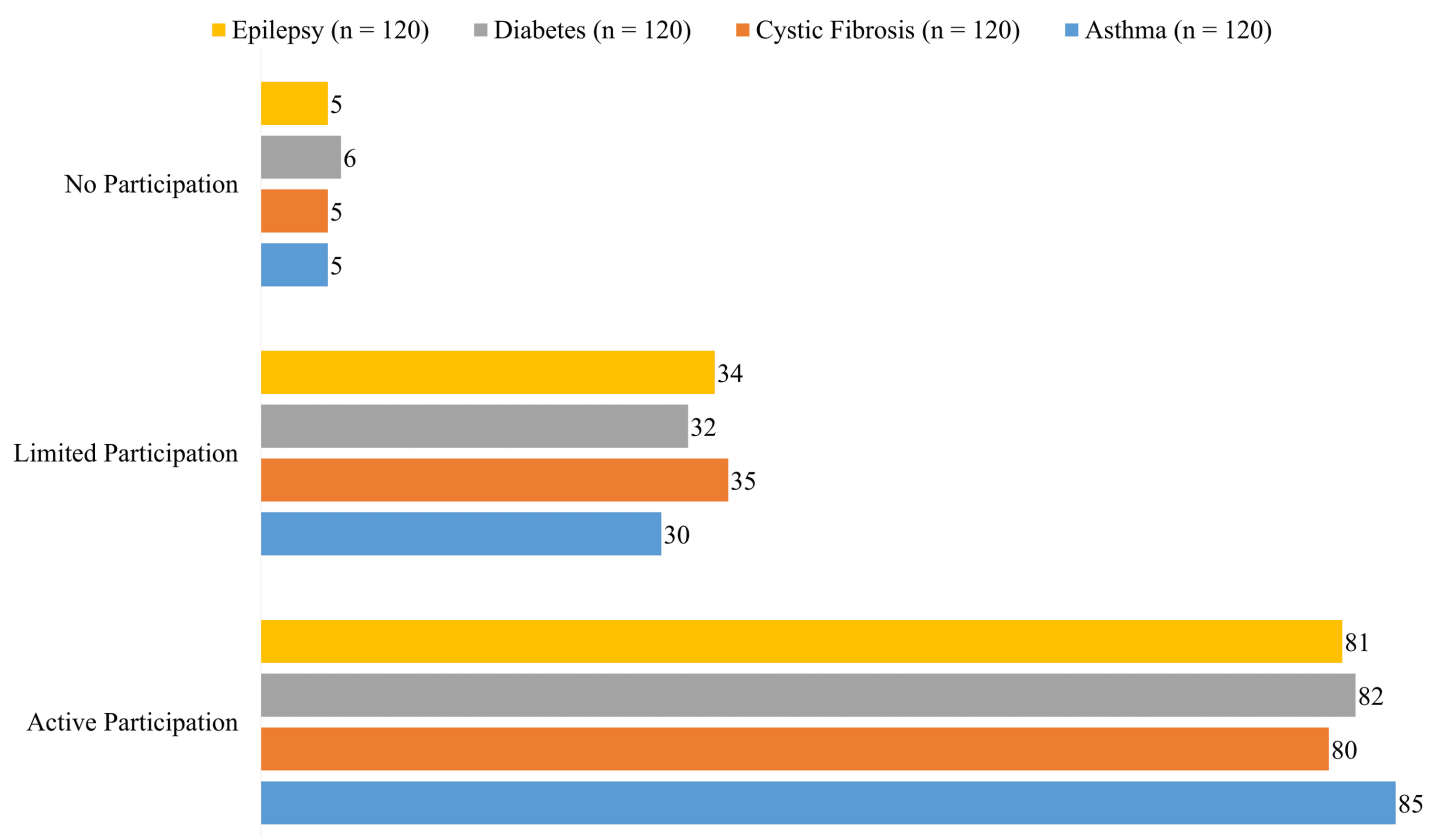
Social activity participation among pediatric patients with asthma, cystic fibrosis, diabetes, and epilepsy.

Children with asthma had the greatest levels of social activity involvement (n = 85), followed by those with diabetes (n = 82), epilepsy (n = 81), and cystic fibrosis (n = 80), as shown in Figure 2. The conditions with the highest rates of limited involvement were cystic fibrosis (n = 35), diabetes (n = 32), epilepsy (n = 34), and asthma (n = 30). All categories had poor participation rates, with diabetes (n = 6) and asthma, cystic fibrosis, and epilepsy (n = 5 each).

The use of technology-driven monitoring and family involvement tactics for four pediatric chronic illnesses is shown in Table 3. A total of 90% of patients with epilepsy, 91.67% of patients with asthma, 95.83% of patients with cystic fibrosis, and 93.33% of patients with diabetes used family education sessions. Additionally, 75% of patients with asthma, 79.17% of those with cystic fibrosis, 76.67% of those with diabetes, and 73.33% of those with epilepsy attended support groups. According to reports, 83.33%, 91.67%, 90.00%, and 87.50% of families participated in care planning. Asthma users used mobile health apps the least (79.17%) and diabetes users the most (87.50%). Remote monitoring was conducted approximately three times a month, with diabetes having the greatest frequency (3.57). In total, 75% of patients with asthma, 79.17% of patients with cystic fibrosis, 77.50% of patients with diabetes, and 76.67% of patients with epilepsy received feedback on their treatment. On average, adherence rate improvements for asthma, cystic fibrosis, diabetes, and epilepsy were 25.09%, 30.19%, 28.32%, and 26.12%, respectively. Asthma, cystic fibrosis, diabetes, and epilepsy all had average overall patient satisfaction ratings of 78.02, 80.03, 82.21, and 79.15, respectively.

**Table 3:** Intervention implementation: family engagement strategies and technology-driven monitoring

Intervention Component		Asthma (n = 120)	Cystic Fibrosis (n = 120)	Diabetes (n = 120)	Epilepsy (n = 120)
Family engagement strategies	Family education sessions (%)	110 (91.67%)	115 (95.83%)	112 (93.33%)	108 (90.00%)
	Support groups (%)	90 (75.00%)	95 (79.17%)	92 (76.67%)	88 (73.33%)
	Family involvement in care planning (%)	100 (83.33%)	110 (91.67%)	108 (90.00%)	105 (87.50%)
Technology-driven monitoring	Use of mobile health applications (%)	95 (79.17%)	100 (83.33%)	105 (87.50%)	98 (81.67%)
	Remote monitoring frequency (times/month)	3.07 ± 1.21	3.22 ± 1.14	3.57 ± 1.08	3.41 ± 1.36
	Feedback provided (%)	90 (75.00%)	95 (79.17%)	93 (77.50%)	92 (76.67%)
Adherence support	Adherence rate improvement (%)	25.09 ± 5.07	30.19 ± 4.52	28.32 ± 5.57	26.12 ± 5.09
Patient satisfaction with interventions	Satisfaction score (scale 0-100)	78.02 ± 10.07	80.03 ± 9.52	82.21 ± 8.05	79.15 ± 9.03

A comparison of changes in hospital admission rates and quality of life ratings across time for juvenile patients with epilepsy, diabetes, cystic fibrosis, and asthma is shown in Table 4. Compared to other conditions, asthma had considerably poorer quality of life ratings at baseline (62.54). By follow-up (T1), all groups had improved: asthma by +12.53, cystic fibrosis by +9.79, diabetes by +10.95, and epilepsy by +10.10. Asthma patients had the greatest incidence of hospital admissions at baseline (4.51), but by follow-up, all groups had significantly decreased, especially those with asthma (−2.45) and cystic fibrosis (−1.61). Diabetes (−0.76) and epilepsy (−1.51) showed smaller declines. Hospital admissions and quality of life score changes were both statistically significant (p < 0.001).

**Table 4:** Comparative analysis of changes in quality of life scores and hospital admission frequencies over time

Outcome Measure		Asthma (n = 120)	Cystic Fibrosis (n = 120)	Diabetes (n = 120)	Epilepsy (n = 120)	P-value
Quality of life score (scale 0-100)	Baseline (T0)	62.54 ± 14.03	66.02 ± 16.05	67.51 ± 14.53	66.28 ± 15.07	p < 0.001
	Follow-up 4 (T1)	75.07 ± 10.52	75.81 ± 12.17	78.46 ± 9.51	76.38 ± 10.09	
	Change in quality of life	+12.53 ± 3.51	+9.79 ± 3.88	+10.95 ± 5.02	+10.10 ± 4.98	
Frequency of hospital admissions	Baseline (T0)	4.51 ± 2.07	3.14 ± 1.59	2.13 ± 1.72	3.35 ± 1.28	p < 0.001
	Follow-up 4 (T1)	2.06 ± 1.37	1.53 ± 1.12	1.37 ± 1.08	1.84 ± 1.42	
	Change in hospital admissions	-2.45 ± 1.12	-1.61 ± 1.08	-0.76 ± 0.64	-1.51 ± 1.11	

## Discussion

The present research investigated the long-term impacts of technology-assisted monitoring and family involvement tactics on the quality of life and hospitalization rates of children with chronic illnesses. All four groups showed substantial increases in quality of life scores: diabetes (67.51 ± 14.53 to 78.46 ± 9.51), cystic fibrosis (66.02 ± 16.05 to 75.81 ± 12.17), epilepsy (66.28 ± 15.07 to 76.38 ± 10.09), and asthma (baseline 62.54 ± 14.03 to 75.07 ± 10.52, p < 0.001). These findings are consistent with other studies that demonstrated the beneficial effects of focused treatments on children's quality of life when they suffer from chronic diseases [16]. Our results of a +12.53 increase in the asthma cohort were supported by the research conducted by Gallefoss et al. [17], which showed comparable improvements in quality of life among asthma patients after a systematic education and treatment program.

We also found a significant drop in hospitalizations, especially among asthma patients, who went from 4.51 ± 2.07 to 2.06 ± 1.37 hospitalizations over the research period (p < 0.001). This drop in hospitalizations is consistent with other research showing that organized intervention programs might cut hospitalizations among children with asthma by as much as 50% [18]. Additionally, our findings demonstrated that hospital admissions for individuals with cystic fibrosis decreased significantly from 3.14 ± 1.59 to 1.53 ± 1.12. This is in line with other studies that found a similar pattern in hospitalization rates after adopting a comprehensive care strategy for treating cystic fibrosis [19].

The benefits of family engagement in care are further supported by our findings on treatment adherence. Asthma, cystic fibrosis, diabetes, and epilepsy all showed high adherence rates (70.83%, 75.00%, 79.17%, and 76.67%, respectively), which are consistent with other studies that found that family involvement in managing chronic diseases greatly raised adherence rates and improved health outcomes [20]. Furthermore, our study's mean gains in adherence rates (25.09 ± 5.07 for asthma and 30.19 ± 4.52 for cystic fibrosis) indicate that consistent assistance and communication greatly enhance adherence.

With scores rising from 55.04 ± 11.58 in asthma to 65.88 ± 8.16 at follow-up, improvements were noted in psychosocial well-being across all conditions, underscoring the psychological and emotional advantages of ongoing family involvement and technology use in the management of chronic illnesses. This result is in line with other studies that highlighted how good family involvement supports psychological well-being and treatment adherence in the management of children's chronic illnesses [21]. When taken as a whole, these findings highlight how important family participation and technology treatments are for improving quality of life and lowering hospitalization rates for kids with long-term illnesses.

Strengths and limitations

Among the study's strengths is its large sample size of 480 children with four distinct chronic illnesses, enabling meaningful comparisons and potentially broad insights. The inclusion of established measures of quality of life and adherence enhances the credibility of our findings, and the longitudinal design allows for the monitoring of changes over time, strengthening the reliability of the results. However, there are several limitations. First, the findings may not be fully generalizable to other pediatric populations, as the study focused specifically on asthma, cystic fibrosis, diabetes, and epilepsy. Additionally, while the study's duration provides valuable insights into medium-term outcomes, a longer follow-up period would be necessary to fully understand the long-term effects of chronic illness management. Finally, while subgroup analysis was not a focus of this study, exploring differences across disease types, age groups, gender, and socioeconomic status would be valuable for future research.

## Conclusions

This research demonstrates how focused family involvement tactics and technology-driven monitoring may significantly enhance quality of life and lower hospital admission rates for children with asthma, cystic fibrosis, diabetes, and epilepsy. The results highlight how crucial it is to treat chronic childhood diseases by combining family engagement with contemporary medical technology. Even though our findings are encouraging, further studies with bigger, more varied populations and control groups are necessary to confirm these therapies and improve care plans for children with long-term conditions.
